# 1-Trifluoromethoxyphenyl-3-(1-Propionylpiperidin-4-yl) Urea Protects the Blood-Brain Barrier Against Ischemic Injury by Upregulating Tight Junction Protein Expression, Mitigating Apoptosis and Inflammation *In* *Vivo* and *In Vitro* Model

**DOI:** 10.3389/fphar.2020.01197

**Published:** 2020-08-07

**Authors:** Xingyang Yi, Chongxi Xu, Pan Huang, Linlei Zhang, Ting Qing, Jie Li, Chun Wang, Tao Zeng, Jing Lu, Zhao Han

**Affiliations:** ^1^ Department of Neurology, People’s Hospital of Deyang City, Deyang, China; ^2^ Department of Neurology, The Affiliated Hospital of Southwest Medical University, Luzhou, China; ^3^ Department of Neurosurgery, West China Hospital of Sichuan University, Chendu, China; ^4^ Department of General Intensive Care Unit, The Second Affiliated Hospital, College of Medicine, Zhejiang University, Hangzhou, China; ^5^ Department of Neurology, Affiliated Hospital of North Sichuan Medical College, Nanchong, China; ^6^ Department of Neurology, Chengdu Fifth People’s Hospital, Chengdu, China; ^7^ Department of Neurology, The Second Affiliated Hospital and Yuying Children’s Hospital of Wenzhou Medical University, Wenzhou, China

**Keywords:** 1-trifluoromethoxyphenyl-3-(1-propionylpiperidin-4-yl) urea, ischemic stroke, blood-brain barrier, tight junction proteins, apoptosis, NF-κB, oxygen-glucose deprivation/reperfusion, inflammation

## Abstract

We previously have revealed that 1-trifluoromethoxyphenyl-3-(1- propionylpiperidin-4-yl) urea (TPPU), as a soluble epoxide hydrolase (sEH) inhibitor can reduce infarct volume, protect blood-brain barrier (BBB) and brain against ischemic injury in rats. Here, we investigated the potential mechanisms of TPPU on BBB integrity in both in permanent middle cerebral artery occlusion (pMCAO) rat model and in oxygen-glucose deprivation/reperfusion (OGD/R)-induced human brain microvascular endothelial cells (HBMVECs) model. In pMCAO rat, TPPU administration decreased brain edema and Evans blue content, increased tight junction proteins (TJs) expression of claudin-5, occludin, and zonula occludens-1 (ZO-1). In OGD/R model, OGD/R significantly increased permeability and cell apoptosis, downregulated the expression of claudin-5, ZO-1, occludin, and lymphoma (Bcl)-2. Notably, TPPU pretreatment effectively protected the BBB integrity by reducing the permeability, promoting expression of claudin-5, ZO-1, occluding and Bcl-2, mitigating reactive oxygen species (ROS) injury and release of interleukin-1β (IL-1β), IL-6β, and tumor necrosis factor-α (TNF-α), downregulating expression of matrix metalloproteinase-9 (MMP-9), MMP-2, bcl-2-associated X protein (Bax), IL-1β, IL-6β, and TNF-α. Moreover, OGD/R induced the up-regulation of p-p65, p-IκB, and p-p38, which were effectively decreased after TPPU pretreatment in comparison with that of the OGD/R group. Furthermore, pyrrolidinedithiocarbamate (PDTC, a selective inhibitor of NF-κB p65) not only alleviated the OGD/R-induced HBMVECs injury and permeability, but also reduced the expression of TNF-α, IL-6, IL-1β, p-p65, and p-IκB, and the protective effect of PDTC was equivalent to that of TPPU. These results indicate that TPPU protects BBB integrity against ischemic injury by multiple protective mechanisms, at least in part, by reducing ROS, inflammation, apoptosis, and suppressing the nuclear factor-κB (NF-κB) and p38 signaling pathways.

## Introduction 

Ischemic stroke (IS) is the leading cause of human neurological disability in China (Guan et al., 2017). Thrombolysis and endovascular thrombectomy are recommended for patients with acute IS (American Heart Association Stroke Council, 2018). However, because of short treatment time window and high risk of bleeding, their clinical use is highly limited (Yaghi et al., 2014). The blood-brain barrier (BBB) is a diffusion barrier between neurons and capillaries, and contributes to the brain homeostasis (Banerjee et al., 2016). Tight junction proteins (TJs), such as occludin, zonula-occludens (ZO), cingulin, and claudin are responsible for the integrity of BBB (Ruck et al., 2014).

BBB dysfunction is one of the pivotal pathological mechanisms of IS (Moretti et al., 2015). TJs play an important role in preventing peripheral leukocyte to the ischemic area and modulating the function of BBB after IS (Krueger et al., 2015). Hypoxia stress can induce the breakdown of TJs, and disruption of TJs impairs the BBB integrity and function (Lv et al., 2018). TJs are important molecular targets for potential drug development and design. Matrix metalloproteinase-2 (MMP-2) and MMP-9 as proteolytic enzymes play key roles in breakdown of BBB following IS by increasing inflammatory response and degrading the extracellular matrix (Zhang et al., 2018). After cerebral ischemia, overproduction of the reactive oxygen species (ROS) not only direct damages neurons, but also contributes to breakdown of TJs and BBB, which result in exogenous large molecules to pass through the barrier into brain and aggravate the brain ischemic injury indirectly (Cheon et al., 2016; Zhang et al., 2017). Apoptosis and inflammation are important causes of BBB disruption and may lead to secondary injury and dysfunction of brain neurons (Sairanen et al., 2006; Tuttolomondo et al., 2014; Kawabori and Yenari, 2015; Cheon et al., 2016). Nuclear factor-κB (NF-κB), as a key regulator for inflammation plays an important role in neuronal injury after IS (Liu et al., 2016). Several studies have demonstrated that p38 mitogen-activated protein kinase (p38 MAPK) signaling pathway was activated after ischemia, leading to upregulate proinflammatory cytokines and neuronal apoptosis (Cai et al., 2011; Xing et al., 2018). Inhibiting p38 pathway can protect against neuronal injury following IS (Zhen et al., 2016). Attenuation of BBB dysfunction is a valuable therapy for reducing ischemic injury (Zhang et al., 2018; Ye et al., 2019; Zhou et al., 2019).

Epoxyeicosatrienoic acids (EETs), as the metabolites of arachidonic acid, exert broadly protective effects against ischemic injury (Jia et al., 2016). However, EETs are rapidly metabolized into dihydroxyeicosatrienoic acids (DHET, less bioactive molecules) by soluble epoxide hydrolase (sEH) (Zeldin, 2001). Our previous studies have revealed that low EETs levels were associated with neurological deterioration and poor prognosis after IS (Yi et al., 2016; Yi et al., 2017). Therefore, sEH is a key enzyme in the EETs metabolic conversion. In the middle cerebral artery occlusion (MCAO) rat model, inhibitors of sEH or gene deletion of sEH can reduce infarct volume (Zuloaga et al., 2015). 1-trifluoromethoxyphenyl-3-(1- propionylpiperidin-4-yl) urea (TPPU) is a sEH inhibitor, which can easily cross the barrier and inhibit expression of sEH in central nervous system (Liu et al., 2013). Recent studies showed that TPPU significantly reduces infarct volume and improves sensorimotor function in MCAO model (Tu et al., 2018). In a myocardial infarction murine model, TPPU has antiapoptosis, antioxidation, antiinflammation, and mitochondrial protection (Sirish et al., 2013). However, the effect and potential molecular mechanisms of TPPU on BBB protection following ischemic injury remain to be investigated.

In this study, we conducted two part trials. First, we evaluated the effect of TPPU on BBB integrity in permanent MCAO (pMCAO) model; Second, we investigated possible molecular mechanisms and signaling modulating pathways of BBB protection of TPPU in oxygen-glucose deprivation/reoxygenation (OGD/R)-induced human brain microvascular endothelial cells (HBMVECs) model.

## Materials and Methods

### Experimental Reagents

TPPU was provided by Dr. Bruce Hammock (University of California, Davis, CA). Dimethyl sulfoxide (DMSO), Evans blue (EB) and fluorescein isothiocyante (FITC)-dextran were all obtained from Sigma Co. (Sigma-Aldrich, St Louise, MA, USA). Human brain microvascular endothelial cells (HBMVECs) were purchased from Angio-Proteomie (Boston, MA, USA). MTT was purchased from Beyotime Biotechnology (Nanjing, China). Phosphate buffer saline (PBS) and fetal bovine serum (FBS) were obtained from PAN-Biotech (Aidenbach, Germany). 2’,7’-dichlorodihydrofluorescein diacetate (DCFH-DA) was purchased from Beyotime Institute of Biotechnology (Shanghai, China). Pyrrolidinedithiocarbamate (PDTC, a selective NF-kB inhibitor) was obtained from Selleck Chemicals (LLC, Houston, TX, United States).

### Rat pMCAO Model and Drug Administration

Adult healthy Sprague-Dawley male rats (6–8 weeks old, 250–280 g) were obtained from Wenzhou Medical University Animal Center. All experiment protocols, including cell culture protocol were reviewed and approved by the Ethics Committee of People’s Hospital of Deyang City and the Institutional Animal Care and the Animal Research Committee of Wenzhou Medical University, all experimental procedures using rats followed the National Institutes of Health Guidelines for the Care and Use of Laboratory Animals.

pMCAO model was established with the intraluminal filament technique as previously described (Zhang et al., 2018). Briefly, Left external carotid artery stump was cut, and a fine surgical nylon monofilament (Jia Ling Biotechnology Co, Guangzhou, China) was gently inserted into left internal carotid artery (ICA) until the blood supply of middle cerebral artery was blocked. In sham group, the animals were treated identically, but the filament was not inserted into ICA. The rats were randomly divided into 5 groups, including sham group, pMCAO group, pMCAO + TPPU (0.5, 1.0, 2.0 mg/kg) group (n=6 for each group). In our pre-experiment, TPPU 1.0 mg/kg was delivered by intraperitoneal injection soon or 2 h after pMCAO, the results showed that brain edema and BBB permeability were lower in TPPU intraperitoneal injection soon after pMCAO compared with 2 h after pMCAO (79.2% in brain edema content and 10.2 μg/g in Evans blue extravasation vs. 85.6% in brain edema content and 14.2 μg/g in Evans blue extravasation, respectively), this indicate that TPPU intraperitoneal injection soon after pMCAO has a better effect. Thus, TPPU at three doses was delivered by intraperitoneal injection soon after pMCAO. The same volume of 5% DMSO was injected intraperitoneally in sham group and pMCAO group (vehicle control). Then, daily intraperitoneal injection of TPPU or 5% DMSO until 3 days before sacrificed.

### Brain Water Content

Wet/dry weight method was used to assess degree of cerebral edema (Zhang et al., 2018). In brief, rats were anesthetized and sacrificed at day 3 after pMCAO. Left and right hemispheres were separated. Left hemisphere (infarct hemisphere) was weighed and then dried for 72 h in an oven of 65°C. Then, dried brain tissue was reweighed. Brain edema content was evaluated according to the formula: brain edema content was (wet weight – dry weight)/wet weight × 100%.

### Evans Blue Dye Leakage Assay

The BBB permeability was evaluated using Evans blue extravasation (Zhang et al., 2018). Briefly, 2 h before sacrificed, the tail vein was injected intravenously 2% Evan’s blue dye at a dose of 4 ml/kg. Then, the rats were transcardially perfused with PBS and sacrificed. The concentration of Evan’s blue dye in the supernatant was assessed using spectrophotometer (610-nm wavelength). The results were showed as Evans blue (micrograms, μg)/brain tissue (g).

### Cell Culture, OGD/R, and Treatment

Primary HBMVECs were continuous cultured in endothelial cell medium (ECM) with 1% penicillin/streptomycin and 10% FBS, and incubated at 37°C and 5% CO_2_. The ECM was replaced by glucose-free medium when the HBMVECs reached 80-90% confluence, and then HBMVECs culture was incubated for 6 h in < 0.5% oxygen in an anaerobic chamber (San Diego, CA, USA). After 6 h of OGD, the complete ECM was used to replace glucose-free medium, and the cells were incubated at 37°C and 5% CO_2_ for 18 h. The HBMVECs were divided into five or six groups: control group, the cells in were maintained in normal culture incubator; OGD/R group, HBMVECs were subjected to 6 h of OGD and 18 h of reoxygenation; OGD/R + TPPU group or OGD/R + PDTC group, the cells were pre-treated with 1.5, 3.0, 6.0 μg/ml TPPU or 100μmol/L PDTC for 2 h before OGD/R (n = 6 for each group), this was based on our pre-experiment. In our pre-experiment, the HBMVECs were pre-treated with 3.0μg/ml TPPU (middle dose) at 2 h before OGD, soon after OGD, and at reoxygenation. The results showed that the cell viability was the highest when cells were pre-treated with TPPU at 2 h before OGD compared with soon after OGD or at reoxygenation, indicating the HBMVECs were pre-treated with TPPU for 2 h before OGD/R has a better effect. Thus, in OGD/R + TPPU group or OGD/R + PDTC group, the cells were pre-treated with TPPU or PDTC for 2 h before OGD/R in this study.

### Endothelial Cell Permeability *In Vitro* Assay

Endothelial permeability was evaluated using FITC-dextran extravasation, as previously described (Chen et al., 2018). Briefly, HBMVECs were seeded onto the collagen-coated inserts. When an endothelial monolayer was formed, the endothelial monolayer was treated with above different treatments. Then, the FITC-Dextran (1 mg/ml) was added on the top of cells, and the cells were incubated at 37°C for 4h. The cell permeability was assessed according to the fluorescence of receiver plate well solution. The fluorescence microplate with an excitation wavelength of 485 and emission wavelength 525 nm was used to evaluate fluorescence intensity, and expressed as percentage of the corresponding normoxic cells.

### Cell Viability Measurement

Cell viability was measured using MTT assay. The cells were cultured in 48-well plates and pretreated with above different treatments for 2 h before OGD/R, then MTT solution (0.5 mg/ml) was added for 4 h at 37°C. The optical intensity was detected by microplate reader (iMark, CA, USA) at 570 nm. The cell viability = absorbance of the treated samples/absorbance of the untreated control × 100%.

### ROS Assay

Cultures were digested with trypsin, cell suspensions were centrifuged at 1,000*g* for 10 minutes, the cells were collected. Single cell suspension was prepared, then DCFH-DA was added to single cell suspension (final concentration of 10 µM). Cells were incubated at 37°C for 30 minutes. The level of intracellular ROS was evaluated using a flow cytometer (Beckman Coulter Epics XL, CA, USA) and analyzed by CELL Quest software. Furthermore, cells were loaded with DCFH-DA at 10 µM for 10 min in dark, then washed using PBS. The cell fluorescence was revealed using fluorescence microscope at emission wavelength of 525 nm and excitation wavelength of 488. Images were photographed in randomly chosen areas using inverted microscope.

### Inflammatory Cytokines Measurement

Cells were washed three times using cold PBS. The supernatant was collected and stored in −20°C. Tumor necrosis factor-α (TNF-α), interleukin-1β (IL-1β) and IL-6β were assessed using ELISA kits (Wuhan Liu he Biotechnology Co., Wuhan, China) according to the manufacturer’s instruction. A microplate reader (Biotek, ELX800, USA) was used to measure optical density at wavelength of 450 nm.

### Western Blotting Analysis

Western blotting was performed to evaluate the expression of associated protein, as previously described (Tu et al., 2018; Chen et al., 2018). Briefly, brain tissues of ischemic penumbra or HBMVECs were lysed and centrifuged at 12,000 *g* for 10 min at 4°C. Total protein was extracted from brain tissues or HBMVECs using protein isolation kit (GE Healthcare, Little Chalfont, UK), the concentration was measured using bicinchoninic assay kit (Thermo Fisher, Massachusetts, Waltham, USA). Proteins (30 μg) were loaded to 10%–12.5% sodium dodecyl sulfate–polyacrylamide gel electrophoresis, then transferred to polyvinylidene fluoride membranes (Millipore Corporation, MA, USA). After blocking with 5% bovine serum albumin for 1.5 h, the membranes were incubated at 4°C overnight with primary antibodies against claudin-5 (1:1,000, Sanying Biotech), ZO-1 (1:1,000, Sanying Biotech, Wuhan, China), occludin (1:1,000, Sanying Biotech), MMP-2 (1:500, Abcam, Cambridge, MA, USA), or MMP-9 (1: 500, Sanying Biotech), lymphoma (Bcl)-2 (1:1,000, Cell Signaling Technology, MA,USA), Bax (1:1,000, Cell Signaling Technology), IL-1β(1:500, Abcam), IL-6β (1:300, Abcam), TNF-α (1:1,000, Abcam), p38 (1:1,000) and phosphorylated p38 (p-p38, 1:1,000) (Santa Cruz Biotechnology, Santa Cruz, CA), p65 and P-p65 (1:1,000, Cell Signaling Technology), IκB and P-IκB (1:1,000, Abcam), GAPDH (1:5000, Santa Cruz Biotechnology). Then, the membrane was incubated using secondary antibody: horseradish peroxidase-conjugated anti-rabbit (1:3,000, Bioworld, Louis Park, MN, USA) at 4°C for 2 h. The immune-reactive bands were determined using chemiluminescence kit and scanned by ChemiDoc XRS imager (Bio-Rad, Hercules, CA, USA), the results were quantified using ImageJ v.7.0 software. GAPDH served as the loading control.

### Statistical Analysis

Statistical analyses were conducted using SPSS 19.0 (SPSS Inc., Chicago, IL, USA). All data are expressed as mean ± standard deviation. Differences among groups were analyzed using analysis of variance (ANOVA), and followed by Student-Newman-Keuls or Tukey *post hoc* test. All analyses were two-sided, and *P* value < 0.05 was defined as statistically significant.

## Results

### TPPU Decreased Brain Edema and BBB Permeability in pMCAO Model

**Figure 1:**
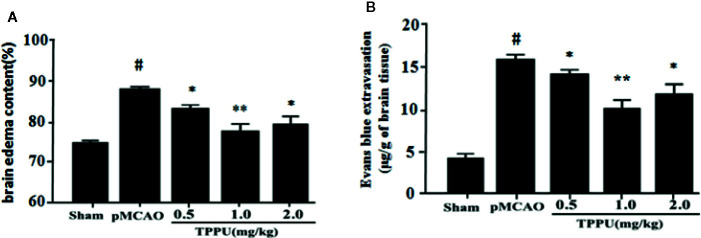
TPPU decreased brain edema and BBB permeability in rat pMCAO model. **(A)** Percentage of brain edema content in the ischemic hemisphere (n = 6 for each group). **(B)** Evans blue dye leakage (n = 6 for each group). ^#^ *P* < 0.01 vs. the sham group; *P < 0.05, **P *<* 0.01 vs. the pMCAO group. pMCAO, permanent middle cerebral artery occlusion; TPPU, 1-trifluoromethoxyphenyl-3-(1-propionylpiperidin-4-yl) urea; BBB, blood-brain barrier.

The degree of cerebral edema was measured using wet/dry weight ratio, and the permeability of BBB was quantitatively assessed by measuring Evans blue extravasation. As expected, rats that underwent pMCAO surgery showed significantly increased brain water content and Evans blue leakage in comparison with the sham group, TPPU significantly decreased the MCAO-induced increase of cerebral edema and Evans blue leakage, with the dose of 1 mg/kg TPPU showing the greatest effect (**Figures 1A, B**). These indicate that TPPU attenuates ischemia-induced BBB disruption.

### TPPU Increased Expression of TJs in Ischemic Brain

**Figure 2:**
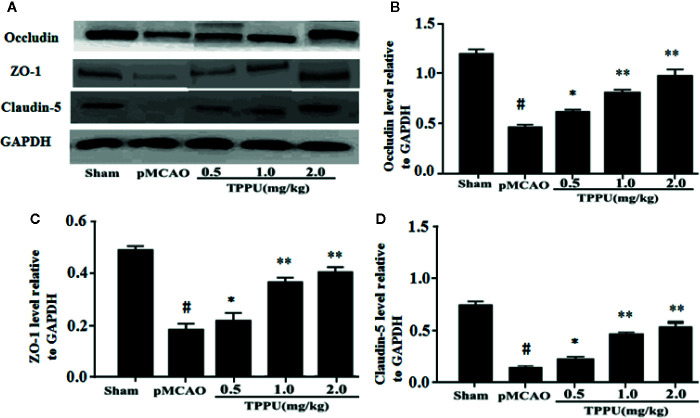
TPPU upregulated the expression of occludin, ZO-1 and claudin-5 in rat pMCAO model. **(A)** Western blotting images. **(B–D)** Densitometric analysis of protein bands (n = 6 for each group). GAPDH served as the loading control. **^#^***P* < 0.01 vs. the sham group; *P < 0.05, **P *<* 0.01 vs. the pMCAO group. pMCAO, permanent middle cerebral artery occlusion; TPPU, 1-trifluoromethoxyphenyl-3-(1-propionylpiperidin-4-yl) urea; ZO-1, Zonula occludens-1; GAPDH, glyceraldehyde-3-phosphate.

TJs are important for the restrictiveness of BBB. For further investigate molecular mechanisms of BBB breakdown, western blotting was used to evaluate the expression of claudin-5, ZO-1 and occludin in brain ischemic penumbra. As presented in **Figure 2**, the expression of claudin-5, occludin and ZO-1 was significantly lower in pMCAO group compared with in sham group (all *P* < 0.01). In comparison with the pMCAO group, the expression of claudin-5, occludin and ZO-1 was significantly increased in TPPU group (**Figures 2A–D**).

### TPPU Alleviated HBMVECs Injury and Permeability After OGD/R Insult

**Figure 3:**
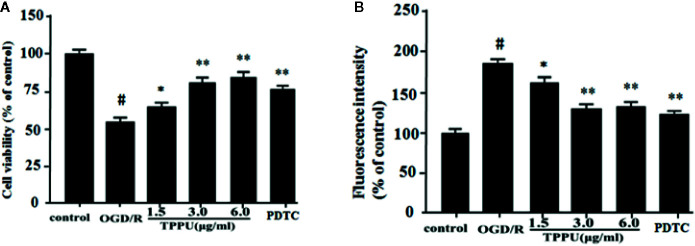
TPPU protected against OGD/R-induced HBMVECs injury and permeability. **(A)** Cell viability was measured in the MTT assay (n = 6). **(B)** HBMVECs permeability. **^#^***P* < 0.01 vs. control group; *P < 0.05, **P *<* 0.01 vs. OGD/R group. OGD/R, oxygen-glucose deprivation/reperfusion; PDTC, Pyrrolidinedithiocarbamate; TPPU, 1-trifluoromethoxyphenyl-3-(1-propionylpiperidin-4-yl) urea; HBMVECs, human brain microvascular endothelial cells.

Then, we investigated the effect of TPPU on OGD/R-induced HBMVECs injury and **permeability**. OGD/R obviously reduced cell survival relative to the control group detected by MTT assay, pretreatment with TPPU significantly decreased OGD/R-induced HBMVECs death, but protection by 6.0 μg/ml TPPU was not significantly different from protection by 3.0 μg/ml (**Figure 3A**). HBMVECs permeability was evaluated using FITC-dextran permeation method. As shown in the **Figure 3B**, HBMVECs permeability was low in control group, but increased in OGD/R group, and noticeably decreased with TPPU intervention. The findings suggest that TPPU treatment may improve OGD/R-induced endothelial barrier function.

### TPPU Inhibited ROS Generation in HBMVECs and Decreased the Level of Inflammatory Cytokines After OGD/R Insult

**Figure 4:**
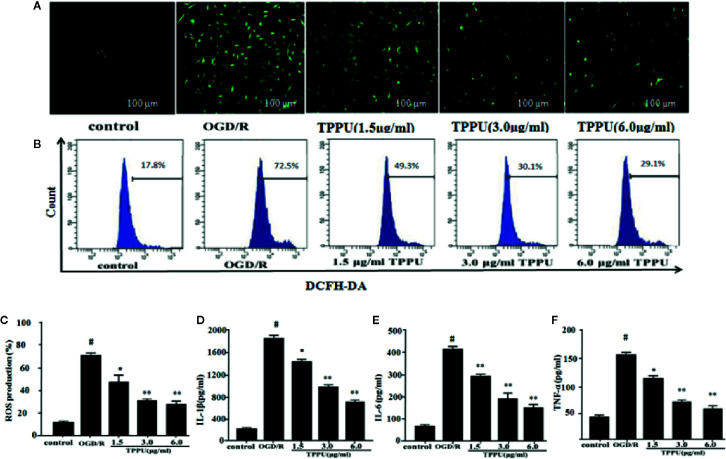
TPPU inhibited the OGD/R-Induced ROS generation and levels of inflammatory cytokines. **(A)** Representative micrographs of ROS generation (under fluorescence microscope). Scale bar = 100 μm. **(B, C)** ROS generation measured by flow cytometry (n = 6). Effects of TPPU treatment on IL-1β **(D)**, IL-6 **(E)**, TNF-α **(F)** (n = 6). ^#^ *P* < 0.01 vs. control group; *P < 0.05, **P *<* 0.01 vs. OGD/R group. OGD/R, oxygen-glucose deprivation/reperfusion; TPPU, 1-trifluoromethoxyphenyl-3-(1-propionylpiperidin-4-yl) urea; ROS, reactive oxygen species; HBMVECs, human brain microvascular endothelial cells; IL-1β, interleukin-1β; IL-6, Interleukin-6; TNF-α, tumor necrosis factor-α.

The BBB damage may be caused by increased oxidative stress and inflammation (Kalogeris et al., 2016). ROS was evaluated by DCFH-DA. The 2’-7’-dichlorofluorescein (DCFH)-positive cells (green) increased in OGD/R group compared to control group, TPPU pretreatment effectively decreased the OGD/R-induced increase of DCFH-positive cells (**Figure 4A**). The level of intracellular ROS was evaluated using flow cytometry (**Figure 4B**). The intracellular ROS level significantly increased in OGD/R group compared with in control group, TPPU pretreatment significantly decreased intracellular ROS level compared to OGD/R group (**Figure 4C**). Furthermore, OGD/R increased the level of TNF-α, IL-6, and IL-1β compared with in control group, however, TPPU treatment significantly decreased the level of these cytokines (**Figures 4D–F**).

### TPPU Decreased Expression of MMPs, Cytokines, and Apoptotic Proteins and Increased Expression of TJs After OGD/R Insult

**Figure 5:**
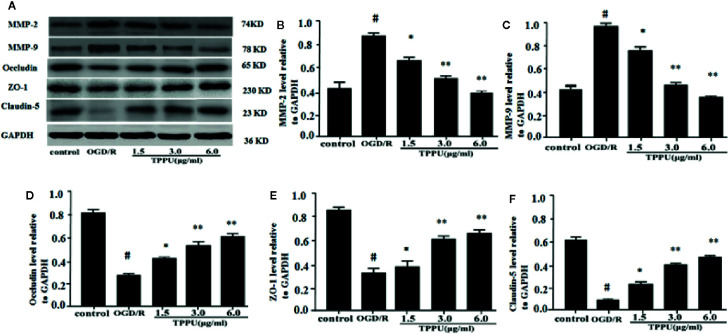
TPPU increased the expression of TJs and decreased the expression of MMPs in OGD/R model. **(A)** Western blotting images. **(B–F)** Densitometric analysis of protein bands (n = 6). GAPDH served as the loading control. ^#^ *P* < 0.01 vs. control group; *P < 0.05, **P *<* 0.01 vs. OGD/R group. TPPU, 1-trifluoromethoxyphenyl-3-(1-propionylpiperidin-4-yl) urea; TJ, tight junction; GAPDH, glyceraldehyde-3-phosphate; OGD/R, oxygen-glucose deprivation/reperfusion; MMP, matrix metalloprotein.

Western blotting analysis showed that the expression of ZO-1, claudin-5, and occludin was lower, and expression of MMP-9 and MMP-2 was higher in OGD/R group than in the control group (**Figure 5A**). TPPU treatment increased expression of Claudin-5, ZO-1 and occludin, and decreased expression of MMP-2 and MMP-9 compared to OGD/R group (**Figures 5A–F**).

**Figure 6:**
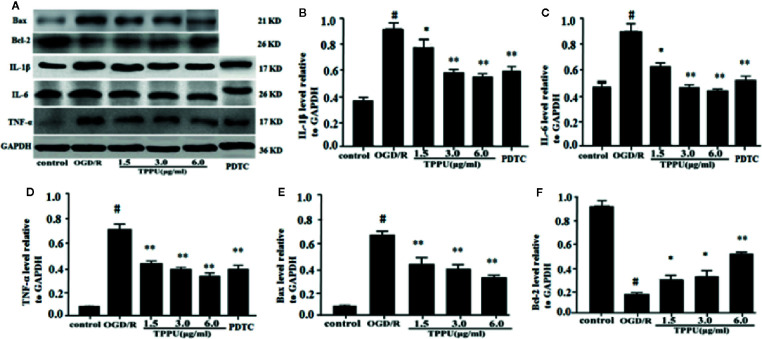
TPPU inhibited expression of inflammatory factors and apoptosis in the OGD/R model. **(A)** Western blotting images. **(B–F)** Densitometric analysis of protein bands (n = 6). GAPDH served as the loading control. ^#^ *P* < 0.01 vs. control group; *P < 0.05, **P *<* 0.01 vs. OGD/R group. TPPU, 1-trifluoromethoxyphenyl-3-(1-propionylpiperidin-4-yl) urea; GAPDH, glyceraldehyde-3-phosphate; OGD/R, oxygen-glucose deprivation/reperfusion; IL-1β, interleukin-1β; IL-6, interleukin-6; TNF-α, tumor necrosis factor-α; PDTC, pyrrolidinedithiocarbamate.

Inflammation and apoptosis play critical role in the brain ischemia-reperfusion injury. Thus, we investigated whether TPPU could effectively inhibit the inflammation and apoptosis after OGD/R-induced HBMVECs. The results were shown in **Figure 6**, the expression of TNF-α, IL-6, IL-1β, and Bax was significantly increased, and Bcl-2 expression was significantly decreased in OGD/R group compared with in control group. After incubation with different concentration of TPPU, the expression of TNF-α, IL-6, IL-1β, and Bax was significantly suppressed, and Bcl-2 expression was significantly increased in comparison with OGD/R group (**Figures 6A–F**).

### TPPU Inhibited OGD/R-Induced Activation of NF-κB and p38 MAPK Signaling Pathways

**Figure 7:**
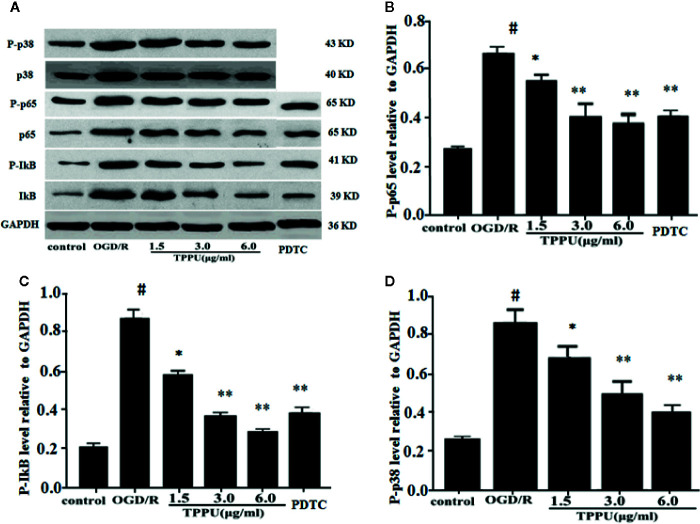
TPPU inhibited OGD/R-induced activation of NF-κB and p38 signaling pathways. **(A)** Western blotting images. **(B–D)** Densitometric analysis of protein bands (n = 6). GAPDH served as the loading control. ^#^ *P* < 0.01 vs. control group; *P < 0.05, **P *<* 0.01 vs. OGD/R group. TPPU, 1-trifluoromethoxyphenyl-3-(1-propionylpiperidin-4-yl) urea; GAPDH, glyceraldehyde-3-phosphate; OGD/R, oxygen-glucose deprivation/reperfusion; NF-κB, Nuclear factor-κB; PDTC, pyrrolidinedithiocarbamate.

To further explore the molecular signaling pathways of neuroprotective effect of TPPU, the effect of TPPU on NF-κB and p38 MAPK pathways were investigated using western blot in OGD/R model. As shown in **Figure 7**, OGD/R induces the up-regulation of p-p65, p-IκB, and p-p38, which were effectively decreased after TPPU pretreatment in comparison with that of OGD/R group (**Figures 7A–D**).

PDTC, as a selective inhibitor of NF-κB p65, was used to further investigate the role of NF-κB signaling in ischemia-induced BBB disruption. The results showed that PDTC not only alleviated the OGD/R-induced HBMVECs injury and permeability (**Figures 3A, B**), but also reduced the expression of TNF-α, IL-6, IL-1β, p-p65, and p-IκB (**Figures 6A–D** and **7A–C**). The protective effect of PDTC was equivalent to that of TPPU. These indicate that TPPU protects BBB integrity against inflammation and apoptosis by inhibiting activation of NF-κB or p38 MAPK signaling pathways after OGD/R insult.

## Discussion

In this study, we investigate potential effect and mechanisms of TPPU on BBB integrity in *in vivo* and *in vitro*. The results demonstrated that TPPU treatment significantly (1) decreased brain edema and BBB permeability, upregulated TJs expression in pMCAO model, (2) inhibited permeability of HBMVECs, decreased the level of ROS and cytokines, increased TJs expression, decreased the expression of inflammatory factors and apoptotic proteins, and downregulated the expression of p-p65, p-p38, and p-IκB in OGD/R model.

BBB permeability is highly selective for macromolecules under physiological conditions. The disruption of the BBB following IS causes brain edema and TJs degradation, direct or indirectly results in damage of brain neurons (Cheon et al., 2016; Zhang et al., 2017). Present study and previous studies demonstrated that sEH inhibitors, such as TPPU, could reduce brain water content and protect the BBB function in MCAO rat model (Zuloaga et al., 2015; Tu et al., 2018). However, we found that the effects of high dose TPPU group (2 mg/kg) on brain edema and Evans blue content were worse than that of middle dose TPPU group (1 mg/kg). This means that the optimal blood concentration of TPPU is 1 mg/kg in our experiment. We speculated that this may be related to the pharmacokinetic of TPPU. TPPU is absorbed rapidly and eliminated slowly, so it can persist in blood longer than other sEH inhibitors because of its metabolic stability (Ostermann et al., 2015). Previous studies showed that concentration of TPPU even at the lowest dose of 0.1 mg/kg was above *in vitro* IC50 values. when the dose of TPPU exceeded 1mg/kg, the EET/DHET ratio was lower compared with 1 mg/kg dose of TPPU (Liu et al., 2013), cautioning against an overdose of TPPU. This may be due to a decrease in the absorption with the increased dose. Meanwhile excessive dose of TPPU would precipitate from the blood due to its limited water solubility (Liu et al., 2013).

However, the mechanisms by which TPPU maintains the BBB functional stability remain unclear. TJs are responsible for restricting permeability and maintaining the stability of the BBB (Krueger et al., 2015). Among TJs, claudin-5, ZO-1, and occludin are very important components for the BBB integrity, yet they are downregulated after IS (Srivastava et al., 2013). Claudin-5 is a sensitive maker of the normal and disturbed states of BBB. Downregulation of claudin-5 can directly lead to increase the BBB permeability. ZO-1 as a TJs regulator, plays a vital role in maintenance of cytoskeleton formation, cell polarity and paracellular barrier. A number of studies have reported that TJs play key role in regulating the integrity and permeability of BBB, downregulating expression of TJs is associated with increase of BBB permeability (Lochhead et al., 2010; Ye et al., 2019). Our present results demonstrated that TPPU treatment could attenuate BBB permeability and increase the expression of TJs, indicating that TPPU protects BBB function against ischemic damage by upregulating expression of TJs.

Although TPPU protects the BBB integrity against ischemia by upregulating expression of TJs, the mechanisms of TPPU regulating TJs remain unclear. Then, we investigated the effect of TPPU on MMP, oxidative stress, inflammation and apoptosis, and found that TPPU significantly inhibited oxidative stress, inflammation, apoptosis and MMP expression in OGD/R model. MMPs can increase inflammatory response and infarct volume, degrade the components of extracellular matrix, and contribute to the BBB breakdown after IS (Lin et al., 2016; Zhang et al., 2018). Previous studies have revealed the interaction between cytokines (TNF-, IL-6, and IL-1β) and HBMVECs triggers inflammation and leads to the increased BBB permeability (Jin et al., 2013; Kalogeris et al., 2016). TPPU can inhibit expression of sHE, increase EETs levels, exert anti-inflammation effect and protect against disruption of BBB (Tu et al., 2018). The findings are consistent with our current results. Oxidative stress may result in the serious brain injury during ischemia (Lochhead et al., 2010; Chuang et al., 2015). ROS production plays a key role in breakdown of BBB in cerebral ischemia/reperfusion (Kalogeris et al., 2016; Li et al., 2019; Ye et al., 2019). In this study, OGD/R-induced significantly increased ROS level in HBMVECs, while TPPU treatment decreased ROS level. Apoptosis is one of main forms of brain ischemia damage and contribute to the BBB impairment (Sairanen et al., 2006; Cheon et al., 2016; Li et al., 2019). Brain ischemia increases ROS generation and inflammatory cytokines, which impair mitochondria, activate the pro-apoptotic protein Bax and stimulate the cytochrome c cascade (Wang et al., 2016). Bcl-2 protein can inhibit downstream apoptotic cascade and block release of cytochrome c (Um, 2016). Upregulation of Bax and downregulation of Bcl-2 have been found after focal brain ischemia in rats (Terashi et al., 2019), and were also found in our current OGD/R-induced HBMVECs model, which are consistent with previous results (Liu et al., 2019; Terashi et al., 2019). Furthermore, TPPU treatment increased Bcl-2 expression and decreased Bax expression, indicating apoptosis was suppressed. Taken together, our findings demonstrate that protective effect of TPPU on TJs, at least in part, can be explained by reduced inflammation, ROS generation, and apoptosis.

Up to date, the signaling pathways of TPPU regulating TJs have not been elucidated. Subsequently, we investigated the possible molecular mechanism of TPPU to regulate NF-κB and p38 signaling pathways. The NF-κB signaling pathway plays an important role in regulating inflammatory response (Liu et al., 2016). Previous studies have shown that inhibition of NF-κB activation can protect brain against further ischemic injury and ameliorate neurological deficit by mitigating TJs disruption and MMP secretion (Su et al., 2017; Nan et al., 2019). In this study, TPPU inhibited the OGD/R-induced NF-κB activation through downregulation of inflammatory cytokines and phosphorylation of p65 protein. Previous study has revealed montelukast may suppress the NF-κB activation through inhibiting nuclear translocation of p65 (Lai et al., 2014). To the best of our knowledge, this is the first study identifying a similar effect of TPPU. Activated p38 MAPK can promote inflammation and apoptosis in both *in vivo* and *in vitro* model and resulting in aggravation of ischemic damage after ischemia (Cai et al., 2011; Xing et al., 2018), and is involved in the BBB dysfunction (Zhang et al., 2017; Li et al., 2019). By western blotting assay in the *vitro* model, the regulatory effect of TPPU on NF-κB and p38 signaling pathways were evident.

Although our findings are interesting, there have several limitations. First, the major aim of current study was to investigate the potential effect and mechanisms of TPPU on the TJs and BBB permeability, we did not evaluate the effects of TPPU on neurological deficits and infarct volume after pMCAO. Second, TJs are complexes, we only evaluated the effect of TPPU on occludin, claudin-5, and ZO-1, other components of TJs, such as ZO-2 and claudin-1 deserved further investigation. Third, signal pathways of regulating inflammation and apoptosis are very complexes, we only evaluated effects of TPPU on NF-κB and p38 signaling pathways. ERK 1/2 and SAPK/JNK sub-family of MAPK signal pathway were not investigated. Fourth, histologic evidences are far more powerful. Our recent study has investigated effect of TPPU on infarct volume, sensorimotor function, BBB protection, expression of TJs, and apoptosis of brain tissue caused by ischemia *in vivo* model (Zhang et al., 2020). In this study, we mainly investigated possible molecular mechanisms and signaling modulating pathways of BBB protection of TPPU in the *vitro* model. Thus, we only evaluated brain water content and the BBB permeability in the *vivo* model. Furthermore, IL-1β, IL-6, TNF-α, and ROS generation were also only measured *in vitro* model. It is better to assess IL-1β, IL-6, and TNF-α secretion *in vivo* to elucidate that TPPU alleviated inflammatory cytokines releasing, and ROS costaining with brain microvascular endothelial cell marker. Thus, further studies are necessary in future.

## Conclusions

In conclusion, TPPU may protect BBB integrity by upregulating expression of TJs against ischemic injury in both *in vivo* and *in vitro* model. TPPU protects effect on TJs against ischemic injury by multiple protective mechanisms, at least in part, by reducing ROS, inflammation, and apoptosis, and inhabiting the NF-κB and p38 signaling pathways. The findings suggest that TPPU may be a promising neuroprotective agent for cerebral ischemia therapies.

## Data Availability Statement

The raw data supporting the conclusions of this article will be made available by the authors, without undue reservation.

## Ethics Statement 

The animal study was reviewed and approved by Ethics committee of People’s Hospital of Deyang City.

## Author Contributions

All authors bear responsibility for the integrity and accuracy of the data in the study. XY and ZH: designed the study and acquired funding. CX, PH, LZ, and TQ: conceived the study and drafted the manuscript. CX, PH, TQ, TZ, and JLi developed the methods and performed experiments. CX, JLu, and TZ analyzed the results and drafted the Figures. XY, ZH, and CW: supervised the project.

## Funding

This study was supported in part by grants from the Sichuan Science and Technology Agency Research Foundation (Grant No.2018JY0164) and the Scientific Research Foundation of Sichuan Provincial Health Department (Grant No. 16ZD046).

## Conflict of Interest

The authors declare that the research was conducted in the absence of any commercial or financial relationships that could be construed as a potential conflict of interest.
